# Synthesis of Bioactive Aminomethylated 8-Hydroxyquinolines via the Modified Mannich Reaction

**DOI:** 10.3390/ijms24097915

**Published:** 2023-04-26

**Authors:** Oszkár Csuvik, István Szatmári

**Affiliations:** 1 Institute of Pharmaceutical Chemistry, University of Szeged, Eötvös u. 6, H-6720 Szeged, Hungary; 2 Stereochemistry Research Group, Eötvös Loránd Research Network, University of Szeged, Eötvös u. 6, H-6720 Szeged, Hungary

**Keywords:** Mannich reaction, 8-hydroxyquinoline, aminomethylation, Betti reaction

## Abstract

8-hydroxyquinoline (oxine) is a widely known and frequently used chelating agent, and the pharmacological effects of the core molecule and its derivatives have been studied since the 19th century. There are several synthetic methods to modify this core. The Mannich reaction is one of the most easily implementable examples, which requires mild reaction conditions and simple chemical reagents. The three components of the Mannich reaction are a primary or secondary amine, an aldehyde and a compound having a hydrogen with pronounced activity. In the modified Mannich reaction, naphthol or a nitrogen-containing naphthol analogue (e.g., 8-hydroxyquinoline) is utilised as the active hydrogen provider compound, thus affording the formation of aminoalkylated products. The amine component can be ammonia and primary or secondary amines. The aldehyde component is highly variable, including aliphatic and aromatic aldehydes. Based on the pharmacological relevance of aminomethylated 8-hydroxyquinolines, this review summarises their syntheses via the modified Mannich reaction starting from 8-hydroxyquinoline, formaldehyde and various amines.

## 1. Introduction

The Mannich reaction allows the formation of a C–C single bond with the involvement of an aldehyde, an amine and a compound possessing a particularly active hydrogen. The essence of the Mannich reaction is that the active H is replaced with an aminomethylene group—if formaldehyde (CH_2_O) is the aldehyde component—or substituted aminoalkyl moiety—if any other aldehyde is applied. During the reaction, a 1-molar equivalent of the H_2_O by-product is formed. The procedure is named after Carl Mannich [1], whose systematic research in this particular field started in 1912 [2]. However, similar condensation reactions were already performed before him, including German patents from 1896 (DE89979) [3] and 1897 (DE90907, DE92309) [4,5] by Bayer & Co. Ltd. (Hong Kong). In the first patent, the procedure included the reaction of dimethylamine, formaldehyde with phenol and naphthol derivatives, as well as the transformation of piperidine, formaldehyde and 1-naphthol. It was suggested that the hydrogen of the phenolic OH group reacts with the aldehyde and amine, resulting in alkylaminomethoxy derivatives. The structures in patent DE92309 were corrected, which thus corresponded to the structures known today as Mannich products. Franz Sachs published his work on the condensation of piperidine, formaldehyde and phthalimide in 1898 [6], and so did Herm Hildebrandt, reporting the condensation of piperidine, formaldehyde, various phenols, and 2-naphthol in 1900 [7]. Mario Betti’s research was launched in 1900 [8,9], in which ammonia, benzaldehyde, and 2-naphthol were reacted. In recognition of his extensive efforts, when a naphthol or phenolic compound is the provider of the active hydrogen, the reaction is referred to as the Betti reaction and the condensation product as the Betti base [10]. Further researchers who also studied this type of condensation before Mannich, are van Marle and Tollens [11], Schäfer and Tollens [12], Auwers and Dombrowski [13], and Petrenko-Kritschenko and his co-workers [14,15,16,17]. In recent times, the procedure has garnered notable consideration due to the versatile nature of its constituents, the use of gentle reaction conditions, and the potential pharmacological activity of the final products [18,19,20,21].

One possible extension of the Mannich reaction is the application of nitrogen-containing naphthol analogues, i.e., hydroxyquinolines (HQ). One of the first bioactive HQs discovered is 8-hydroxyquinoline (8HQ), which itself is a well-known antipathogenic and chelating agent [22]. It has many derivatives with more or less similar properties, including clioquinol, chlorquinaldol, chloroxine, broxyquinoline, iodoquinol, nitroxoline, and tilbroquinol [23,24,25,26,27,28,29]. In contrast, procaterol is a β2-adrenoreceptor agonist used in the treatment of asthma [30]. The Mannich derivatives of 8HQs have a prominent place in medicinal chemistry [31] and have been reviewed from many angles [1,18,19,20,22]. In this framework—covering more than one century of Mannich chemistry—derivatives in which the 8HQ and amine functions are linked by a methylene bridge will be included because aminoalkylation of 8HQs has not been reviewed from this particular chemical perspective. These compounds are synthesised by treating the 8HQ core with formaldehyde (paraformaldehyde or formalin, its aqueous solution) and an amine. These derivatives also possess diverse pharmacological activities. The antipathogenic effect is one of the most studied areas, including different mechanisms of action: increasing cell membrane permeability [32], inhibition of MetAP1 [33,34], ubiquinone synthesis [35], or type III secretion [36,37]. Antifungal activity of these 8HQs has been assessed among humans [34,38,39] and phyto-pathogens [40,41]. Furthermore, a potential antiprion compound has also been identified [42]. Clamoxyquine is an effective drug for treating whirling disease in rainbow trout [43]. Recently, a large group of aminomethylated 8HQs has been designed to combat cancer. There are derivatives that act by inhibiting DNA biosynthesis [32,44], MetAP2 [45], JMJD2C [46], and Rcel [47]. MDR-targeting agents have been studied as well [48,49], while in some derivatives, the metal-binding property has been utilised to enhance the antiproliferative activity [50,51,52,53]. The effects on the MAPK pathway [54], the caspase-dependent apoptotic pathway [55,56] and survivin [57,58] have also been examined, while some 5-nitro compounds were associated with inhibition of cathepsin B [59,60,61,62]. In the last few years, potential neuroprotective agents have been identified to treat Alzheimer’s disease by influencing multiple intramolecular targets [63,64,65,66]. Concerning the central nervous system, dopamine D2 receptor [67,68] and inward rectifier potassium channels [69] have also been targeted with 8HQ Mannich products.

## 2. Syntheses of Aminomethylated 8-Hydroxyquinolines

The next sections are divided considering two aspects: first, the ratios of the incorporated reagents in the Mannich product (CH_2_O, amine and 8HQ) and second, the structure of the applied amine and 8HQ.

### 2.1. Syntheses of Mannich Bases Furnishing 1:1:1 (CH_2_O:Amine:8HQ) Ratio in the Product

This subsection will be organised with regard to the order of amines and their structure (primary, acyclic and cyclic secondary).

#### 2.1.1. Syntheses by Using Primary Amines

Various primary amines were reacted with 8HQ (**1**) and formaldehyde (CH_2_O), resulting in the formation of Mannich bases **2**–**13** (Table 1). Compound **2** was synthesised by Zaoui et al. by treating **1** with an aqueous solution of CH_2_O (37%; hereinafter formalin) and octylamine in EtOH [70]. Fields carried out the synthesis of **3a**,**b** in two steps: first, an azomethine was obtained from CH_2_O and the corresponding amine, and subsequently, it was stirred with **1** in benzene (**3a**) or without solvent (**3b**) [71]. Burckhalter et al. used *N*^1^,*N*^1^-dimethylethane-1,2-diamine and paraformaldehyde to transform **1**. After stirring the components in EtOH and then removing the solvent, the mixture was treated with hydrogen chloride gas in excess, isolating **4** with excellent yield [72]. Xie and Ding reacted 3-methylbutan-1-amine or 2-morpholinoethylamine with **1** and paraformaldehyde for 4 h under reflux conditions, resulting in **5a**,**b** [46]. The synthesis of **6a**–**c** was performed by Manetti et al. by applying **1**, paraformaldehyde and the appropriate amine [73]. The synthesis of various Mannich products was published by Mohammed et al., including 7-((phenylamino)methyl)quinolin-8-ol (**7**), applying paraformaldehyde as the source of CH_2_O (for conditions, see Table 1) [47]. The synthesis of thiourea derivative **8** was carried out by Abuthir et al. [74]. Benzensulfonamide derivative **9** was furnished by Shaw et al. by dissolving **1**, paraformaldehyde and the amine in dry EtOH at room temperature (r.t.) and then treating the mixture under reflux conditions [55]. **10a**–**c** were synthesised by Banerjee et al. by stirring CH_2_O with the appropriate amine in EtOH. After the addition of **1**, the mixture was cooled on ice, followed by a pH adjustment to 5–6 [75]. **11a**–**g** were prepared by Tripathy et al. (**11a**), Sahoo et al. (**11b**,**c**,**g**) and Bhargava and Sharma (**11d**–**f**) by means of stirring **1**, 4-substituted-2-aminothiazole and paraformaldehyde in the presence of HCl in EtOH [76,77,78]. Novel triheterocyclic systems (**12a**–**m**) were described by Mallur et al., applying reaction conditions similar to those of Sahoo et al., Tripathy et al. and Bhargava and Sharma [79]. Fernández-Bachiller et al. reported tacrine−8HQ hybrids (**13a**–**g**), synthesised via heating paraformaldehyde and the corresponding diamine at 90 °C in EtOH. The mixture was then cooled, and 8HQ, dissolved in EtOH, was added dropwise, followed by stirring at r.t. [64].

**Scheme 1:**
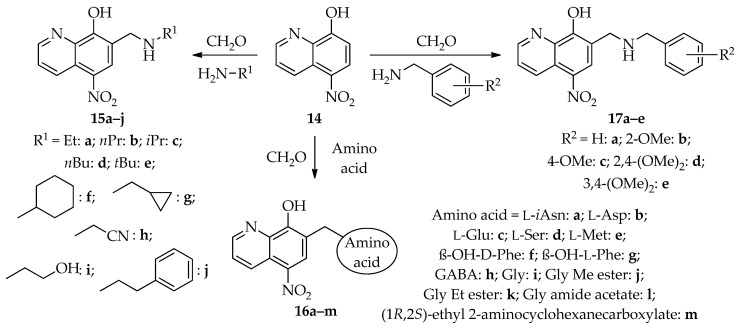
Reaction of 5-NO_2_-8HQ (**14**), CH_2_O and primary amines including primary alkyl amines, substituted benzyl amines and amino acids.

The 7-aminomethylation of nitroxoline (5-nitro-8HQ, **14**) is achieved by using different primary amine sources: aliphatic amines, amino acids and substituted benzylamines (Scheme 1). Sosič et al. synthesised various derivatives, including **15a**, **15h**, **16m** and **17a**, by heating **14** in pyridine and then adding formalin (≥36.5%) and the desired amine [60]. Yin et al. carried out the synthesis of **15b**–**g** in dry EtOH under reflux conditions [40]. One of the first to deal with the Mannich bases of **14** was Movrin et al., who synthesised **15i** and **15j**, reacting **14**, paraformaldehyde and the corresponding amine in pyridine [80]. Morvin and Marok also tested various amino acids in the Mannich reaction, providing **16a**–**l** [81]. Szakács et al. furnished **17b**–**e** by heating the mixture of **14**, formalin (37%) and substituted benzylamines in pyridine at reflux temperature (50 °C) [48].

Primary amines applied in 5-chloro-8HQ (**18**) transformations include aliphatic amines, diamines and benzylamines (Table 2). Burckhalter et al. did not only synthesise **4**, but they also prepared its 5-Cl derivative **19a** and also **20a** in a similar way [71]. In addition, Burckhalter also provided further compounds (**19b**, **20b**, **21**, **22**, **23c**, **24a**, **24b**, **25**, **27b**, **31**, **32**) by treating **18** with paraformaldehyde and appropriate amines in EtOH under reflux conditions for 90 min, followed by a treatment with hydrogen chloride [82]. One exception is **27a**. In this case, the mixture was heated until a thick, oily material remained. Later, **20b** proved to be an efficient antiamoebic and antidiarrheal agent and became known as clamoxyquine [43,83,84]. Burckhalter et al. prepared **20c**–**e**, **23a**, **23b**, **26**, **34**–**36**, using the appropriate amine and CH_2_O in the form of paraformaldehyde [85]. Bolognesi et al. synthesised compound **28** by carrying out the reaction in toluene and using paraformaldehyde [42]. Fernández-Bachiller et al. applied not only **1** as the starting scaffold for tacrine–8HQ hybrid but also **18**, yielding **29a**–**e** [64]. **33a**,**b** was furnished by stirring **18**, the corresponding amine and 1.1 equivalents of paraformaldehyde in absolute EtOH at 60 °C for 16 h by Ahn et al. [63]. If 2 molar equivalents of CH_2_O were applied in the synthesis of **33a**, the concomitant formation of benzoxazine **30a** was also observed. Note that the synthesis of other benzoxazines will be discussed in Section 2.2 [63]. Kenyon et al. published the preparation of **37** using other Mannich bases. The starting compounds were stirred without solvent at 120–150 °C; therefore, the mixture melted, forming the desired products [86]. Szakács et al. described the synthesis of **38** and **39b**–**d** from **17b**–**e** but applied different conditions. To obtain **38** and **39c**,**d**, the EtOH solution of **18** mixed with cyclohexylamine and formalin solution was stirred under different conditions: 4 days at r.t. (**38**), 14 days at r.t. (**39c**), and 120 h at 50 °C (**39d**) [48]. **39b** was prepared from benzoxazine **30b** under acidic conditions (HCl/EtOH 22%) in 1 h at r.t. [48]. Compound **39a** was synthesised by Combes and Mesnier via sulfuric acid treatment of **30c**, which was previously prepared [87,88]. Gianni et al. reported the formation of **39e**, after treating **18** with formalin and phenethylamine in MeOH at r.t. for 12 h [89].

**Scheme 2:**
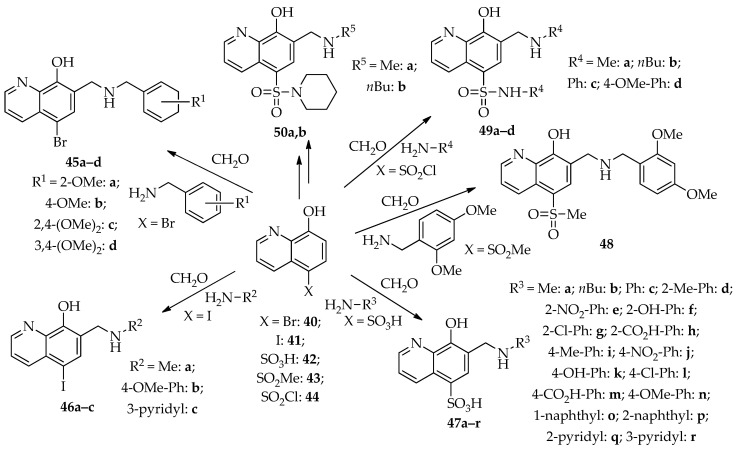
Reaction of 5-substituted 8HQs, CH_2_O, and primary amines.

Scheme 2 shows the aminomethylation of additional 5-substituted 8HQs (**40**–**44**). Szakács et al. transformed 5-bromo-8HQ (**40**) to **45a**–**d** upon stirring **40** with formalin (37%) and the corresponding amine for 1 h [48]. Mannich reaction of **41** was performed by Yanni, testing methylamine, *p*-anisidine and 3-aminopyridine, thus acquiring **46a**–**c** [90]. The substitution of 8-hydroxyquinoline-5-sulfonic acid (**37**) at C-7 with various alkyl- and arylamines was reported by Yanni and Timawy (**47a**–**r**) [91]. **48** was also delivered by Szakács et al. by stirring 2,4-dimethoxybenzylamine dissolved in EtOH, formalin (37%) and **43** at 60 °C for 48 h [48]. Yanni et al. accomplished the concomitant formation of the 7-aminomethyl- and 5-sulfonamide functional groups starting from **44**, paraformaldehyde and the corresponding amine. The reaction mixture was treated under reflux conditions in EtOH, yielding **49a**–**d**. Subsequently, they carried out sulfonamidation and then aminomethylation of **39**, giving **50a**,**b** [92].

The Mannich transformation of various 8HQs (**51**–**59**) listed in Table 3 was performed under rather similar conditions, despite the fact that they were carried out by different research groups. Fernández-Bachiller et al. prepared **60a**–**e** utilising exactly the same conditions used in the fabrication of **13a**–**g** and **29a**–**e** [64]. **61a**–**c** were synthesised by Bourquin et al., applying 1-methylpiperidine as an amine [93]. The transformation of 4-chloro-2-methylquinoline-8-ol (**54**) and 4-chloro-3-(2-chloroethyl)-2-methylquinoline-8-ol (**55**) was performed by Ozawa and Shibuya, resulting in **62a**,**b** [94]. In addition to **6a**–**c**, Manetti et al. also synthesised **63a**,**b** from 4-butoxy (**57**) and 4-benzyloxy (**58**) derivatives [73]. The preparation of **64a**,**b** was reported by Shoeb et al. using paraformaldehyde under the conditions indicated [95]. Yanni and Mohharam reacted the 7-sulphonic acid derivative of 8HQ with various aromatic and aliphatic amines and paraformaldehyde in the Mannich reaction, which afforded the formation of compounds **65a**–**k** [96].

**Scheme 3:**
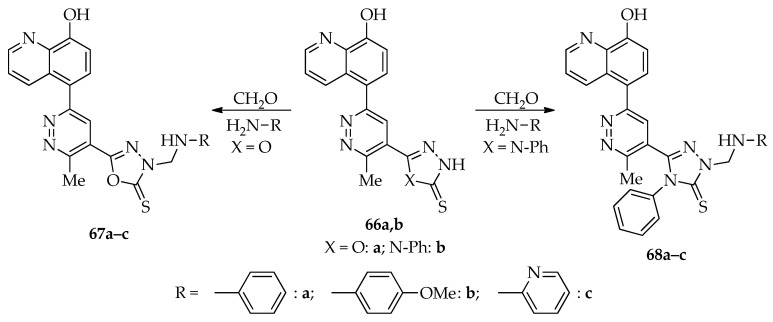
Reaction of 5-substituted 8HQs, CH_2_O, and primary amines.

The transformation of pyridazine-annulated 8HQ was implemented by Abdelmohsen, who treated the solution of **66a** or **66b** in abs. EtOH with formalin (40%) and then added the aromatic amine in EtOH. Subsequently, the mixture was stirred for 3 h at r.t. and left overnight with the result of **67a**–**c** and **68a**–**c**. An interesting feature is that the Mannich reaction occurred on the side chain rather than on the 8HQ core at position 7 (Scheme 3) [97].

#### 2.1.2. Syntheses by Using Acyclic Secondary Amines

The results of aminomethylation of 8HQ and 5-nitro- and 5-halogeno-8HQs with symmetric and asymmetric acyclic secondary amines are listed in Table 4. One of the earliest utilisations of the Mannich reaction to transform 8HQ (**1**) was performed by Burckhalder et al. when equimolar amounts of dimethylamine, paraformaldehyde and **1** were dissolved in EtOH and treated under reflux for 2 h (**70a**, yield: 74%) [98]. Philips and Fernando prepared **70b** by mixing paraformaldehyde and diethylamine, then adding **1** dissolved in EtOH and, after a one-hour standing, the mixture was treated under reflux for 5 h [99]. Motaleb et al. used DMF as a solvent and paraformaldehyde as the aldehyde source to synthesise dicarboxylic acid derivative **70c** [100]. The *n*-propyl derivative **70d** was prepared by Faydy et al. in two steps: first, the Mannich reagent was synthesised by mixing dipropylamine and paraformaldehyde in EtOH, and in the second step, 8HQ in EtOH was added. Subsequently, after 1 h at r.t., the mixture was treated at reflux for 3 h [101]. The synthetic process of **70e**–**q** was performed by Ishida and Watanabe. Formalin (35%) was added dropwise to the mixture of **1** followed by the addition of the ethanol solution of different amines. Then, the reaction mixture was stirred for 1 h at r.t. and kept under reflux for 3 h [102]. Szakács et al. also used formalin (37%) to furnish **70r** [48]. **71a** was the product of the reaction of **14** with dimethylamine and paraformaldehyde in pyridine/EtOH under reflux conditions, performed by Shterev et al. [103]. **71b** was obtained by Burckhalder et al. by dissolving the components (**14**, paraformaldehyde and diethylamine) in EtOH and heating at reflux for 90 min [104]. Yin et al. reported dipropyl homologue derivative **71c**, while Movrin et al. described the *i*Pr and (CH_2_)_2_OH derivatives (**61d**,**e**) [40,80]. Liu et al. performed the synthesis of **71f** by dissolving **14** and CH_2_O in EtOH, then adding dicyclohexylamine and treating the mixture under reflux for 24 h [41]. The synthesis of compound **71g** was disclosed by Elofsson, and acetonitriles **71h**,**i** were prepared by Sosič et al. [36,60]. Helin and Vanderwerf synthesised **72** by adding paraformaldehyde and diethylamine in EtOH to the 1:1 ether:EtOH solution of 5-fluoro-8HQ (**69**) and leaving it to stand for 30 min [105]. The aminomethylation of **18** and **40** leading to the formation of **73** and **74** was studied by Burckhalter et al. [71,85,106,107].

Table 5 shows further 5-substituted 8HQ derivatives. The synthesis of **96a**,**b** was performed by Edgerton and Burckhalter by adding **75** in EtOH to the heated EtOH solution of the appropriate amine and paraformaldehyde and exposing it to 1–2 h of reflux [108]. **97** was furnished by mixing **76**, paraformaldehyde and dibenzylamine in abs. EtOH, and treating it under reflux for 4 h by Himmi et al. [109]. Venkataramani applied paraformaldehyde as a CH_2_O source in the reaction of **77** and **79** with ethanolamine, which yielded **98** and **100** [110]. **99** was synthesised by Burckhalter and Leib by treating the EtOH solution of the components (**78**, paraformaldehyde and diethylamine) at reflux for 3 h [111]. Schraufstätter and Bock extended the reaction to several 5-acyl derivatives, delivering **101a**, **102a**–**e** and **103**–**109** by stirring the mixture of **80**–**93** each with formalin (30%) and the appropriate amine in EtOH at reflux [112]. **101b** was synthesised by Mangeney and Pechmèze by reacting **80**, formalin (30%) and the amine at reflux for 10 h [113]. **102f** was obtained by Gopalchari and Dhar by stirring the starting compounds (**83**, paraformaldehyde and diethylamine) in EtOH at reflux [114]. Möhrle and Schaltenbrand gained epoxy ketone **94** from **87**, and subsequently **110a**,**b** from **94** [115]. **111** was synthesised by Burckhalter et al. from **95** with a yield of 74% [104]. Sen and Kulkarni carried out the transformation of **42**, applying paraformaldehyde and several dialkyl amines, which gave products **112a**–**f** [116]. Yanni et al. treated **44** with diethylamine and paraformaldehyde, leading to the formation of **113** [92].

**Scheme 4:**
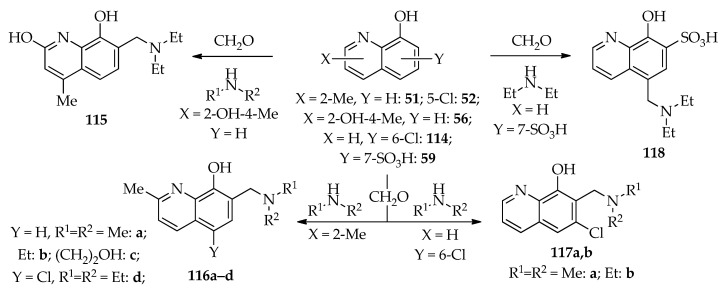
Reaction of 8HQ derivatives, CH_2_O, and acyclic secondary amines.

Scheme 4 includes further aminomethylated 8HQs. In addition to the preparation of **64a**,**b**, the synthesis of **115** was also carried out by Shoeb et al. [95]. The dimethyl and diethyl derivatives of quinaldine (**116a**,**b**) were first reported by Bourquin et al., and then the synthesis of **116d** was shown to be efficient from both **52** via the Mannich reaction and **116b** via chlorination [93]. **116c** was furnished using diethanolamine and CH_2_O by Ozawa and Shibuya, with the finding that the reaction occurred at position 7, not involving the 2-Me group [117]. The transformation of 6-chloro-8-hydroxyquinoline (**114**) to **117a**,**b** was performed by Burckhalter et al. Their interest was motivated by the biological importance of these isomeric structures (**73a**,**b**) [71,107]. The synthesis of **117a** was performed in EtOH, but the synthesis of **117b** could be carried out efficiently in both MeOH and EtOH using paraformaldehyde as the CH_2_O source. Yanni and Mohharam prepared **118,** similar to the method applied in the synthesis of primary amine derivatives **65a**–**k** [96].

#### 2.1.3. Syntheses by Using Cyclic Secondary Amines

**Scheme 5:**
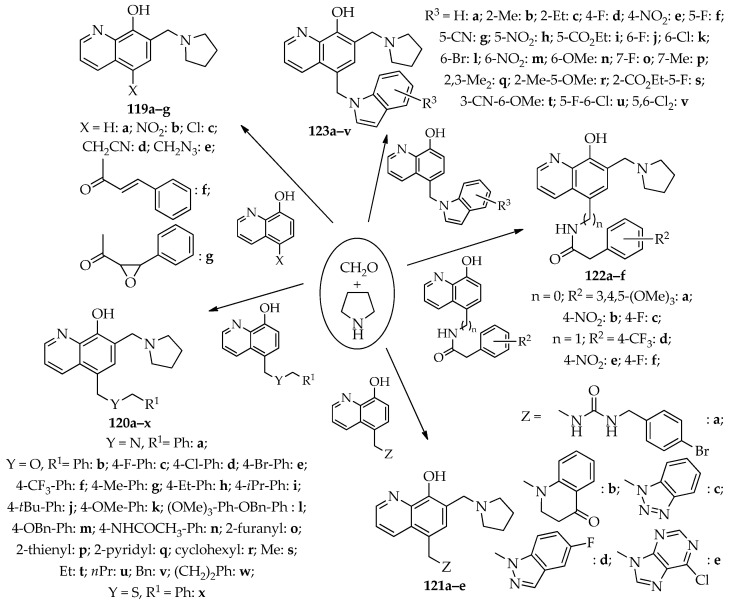
Reaction of 8HQ and substituted 8HQs, CH_2_O and pyrrolidine.

This subsection covers Mannich reactions carried out with the use of cyclic amines. Scheme 5 depicts the transformation of 8HQ derivatives by pyrrolidine and CH_2_O. **119a**, the simplest core, was reported by Goyal and Chaturvedi [118]. The synthesis of **119b**–**g** is an extension of methods described previously by Movrin et al. (**119b**) [80], Burckhalter et al. (**119c**) [71], Himmi et al. (**119d**,**e**) [109], Schraufstätter and Bock (**119f**) [112] and Möhrle and Schaltenbrand (**119g**) [115]. Numerous 5-substituted 7-pyrrolidinylmethyl derivatives (**120a**, **120c**–**x**) were obtained by Xiao et al. on the basis of **120b** (UC-112) by reacting the appropriate functionalised 8HQs with paraformaldehyde and pyrrolidine in EtOH [57]. Note that this approach was previously studied by Wang and Li [56]. Based on these findings, the synthesis of further UC-112 analogues (**121**–**123**) was carried out under the same conditions by Wang et al. [58].

**Scheme 6:**
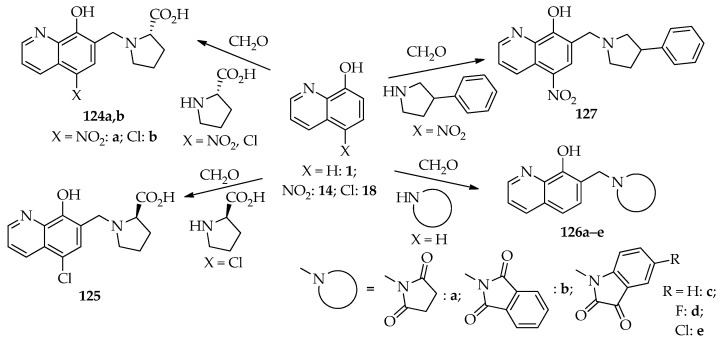
Reaction of 8HQ and substituted 8HQs, CH_2_O and pyrrolidine derivatives.

Scheme 6 shows the application of functionalised/condensed nitrogen-containing five-membered ring systems. The utilisation of L- and D-proline in the Mannich reaction was described by Mészáros et al. and the Pivarcsik group. Compounds **124a**,**b** and **125** were synthesised by stirring L- or D-proline with formalin (38%) and the appropriate 8HQ (**14** or **18**) in MeOH at 75 °C [51,52,53]. Further derivatives were described by Mohamed et al. and Elofsson et al., including phthalimide, isatin and 5-halogenoisatins, succinimide and 3-phenylpyrrolidine affording **126a**–**e** and **127** [36,119].

Piperidine, a secondary amine, has been frequently utilised in the Mannich reaction (see Table 6), which is probably due to its stability and reactivity. Note that it was the first amine to be applied for the aminomethylation of 8HQ, described in a German patent in 1897 [5]. Briefly, 14 kg of 8HQ (**1**) was dissolved in EtOH, then 8 kg of formalin (40%) and 8.5 kg of piperidine were added, and the mixture was stirred for 6 h under reflux. After distillation, the free base (**137**) was crystallised. Its hydrochloride salt was prepared by Burckhalter et al. [97]. The reaction conditions for the compounds in Table 6 are almost identical. EtOH was used as a solvent, except for compound **144**, which was performed in a solventless process by Burckhalter and Leib (for **146a**,**b**, **148d** and **150,** details were not available) [109,111,115]. Reaction mixtures were treated primarily at reflux or at an unspecified heated temperature, with the exception of **145**, synthesised at r.t. [48]. Additional examples are **138**, **139**, **151**, **152** (Burckhalter et al.) [71,104]; **140**, **147** (Burckhalter and Leib) [111]; **141** (Yanni) [90]; **142a**–**c**, **148a**–**c** (Edgerton and Burckhalter) [108]; **143a**,**b** (Himmi et al.) [109]; **146c** (Xiao et al.) [57]; **149** (Schraufstätter and Bock) [112]; **153** (Sen and Kulkarni) [116] and **154** (Yanni) [92]. In the case of **154**, reactions started from **44**, and not only aminomethylation but also concomitant sulfodamidation was achieved.

**Scheme 7:**
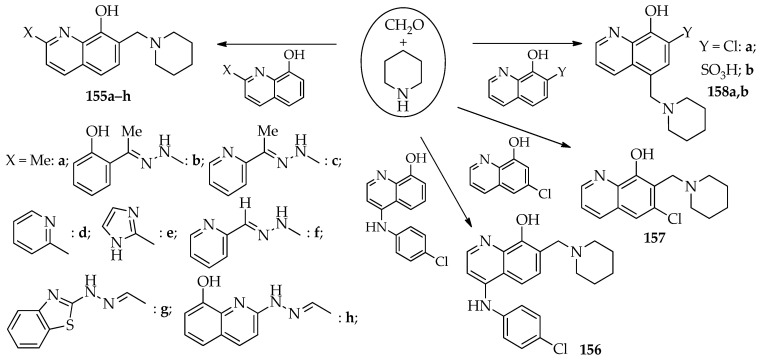
Reaction of 2-, 4-, 7- or 8-substituted 8HQs, CH_2_O and piperidine.

The transformations of 2-, 4-, 7- or 8-substituted 8HQs are depicted in Scheme 7. Quinaldine **155a** was obtained by reacting equimolar amounts of 8-hydroxyquinaldine, paraformaldehyde and piperidine in EtOH for 3 h under reflux conditions by Rose et al. [120]. Szakács et al. transformed some 2-functionalised 8HQs by stirring the mixture of piperidine and formalin (35%) in EtOH for 1 h, then, after adding the appropriate 8HQ, stirring was continued at r.t. for 2 days (**155d**), 3 days (**155e**–**h**) or 4 days (**155b**,**c**) [48]. 4-(4-Chloroanilino)-8-hydroxyquinoline was added to the heated ethanolic solution of piperidine and paraformaldehyde and heated to boiling for 25 min to obtain **156** by Burckhalter and Edgerton, who also synthesised **157** in 72% [106]. To provide **158a**, 7-chloro-8-hydroxyquinoline, piperidine and paraformaldehyde were dissolved in MeOH and heated at reflux for 90 min [71]. Among the 8-hydroxyquinoline-7-sulfonic acids previously described, **158b** was reported by Yanni and Mohharam [96]. 

**Scheme 8:**
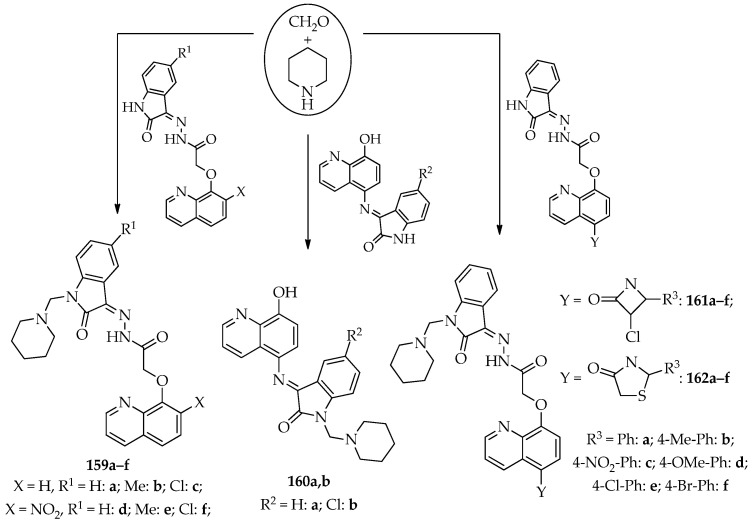
Reaction of substituted 8HQs, CH_2_O and piperidine.

Additional piperidylmethylene-substituted 8HQs are included in Scheme 8. Compounds **159a**–**f** were synthesised by Meenakshi et al. [121], and derivatives **160a**,**b** were published by Chhajed and Padwal [122]. **161a**–**f** and **162a**–**f** were prepared by Madhu et al. The starting 8HQ, piperidine and water were mixed, and after adding H_2_O and DMF to this clear solution, stirring was continued in an ice bath for 2 h, followed by leaving at r.t. overnight [123,124]. Similar to **67a**–**c** and **68a**–**c**, these are exceptional cases, since piperidine and formaldehyde did not appear to react with the 8HQ nuclei but resulted in *N*-functionalisation of the isatin core.

**Scheme 9:**
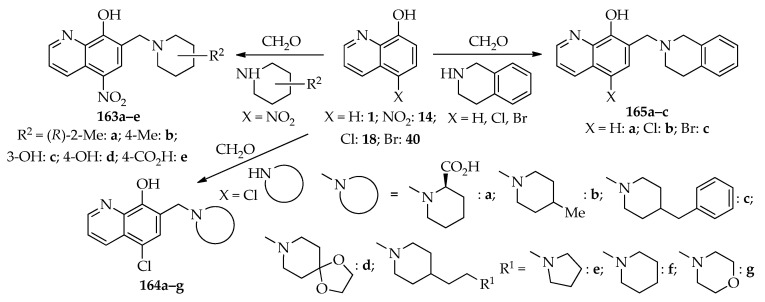
Reaction of 8HQ and 5-substituted 8HQs, CH_2_O and piperidine derivatives.

Scheme 9 depicts the Mannich reaction of 8HQs with various functionalised/condensed piperidine derivatives. **163a** was synthesised by dissolving **14** in pyridine at 60 °C, adding formalin (36.5%) and (*R*)-2-methyl-piperidine, and then stirring until the reaction was complete [60]. The synthesis of compound **163b** was disclosed by Mirković et al. [59]. **163c**–**e** were prepared by Shterev et al. by treating **14** with piperazine derivatives and paraformaldehyde in a 1:1 mixture of pyridine and EtOH [103]. In addition to prolines, Pivarcsik et al. also applied (*R*)-piperidine-2-carboxylic acid (L-pipecolic acid), resulting in **164a** under the same conditions used for **124a**,**b** and **125** [52]. Only a few examples were reported on the utilisation of microwave irradiation (MW) in the Mannich reaction of 8HQ. In one of them, demonstrated by Swale et al., 20–30 min of MW irradiation at 140 °C and EtOH as solvent were applied to gain **164c**–**d** [69]. Compounds **164e**–**g** were obtained by Chough in the reaction of **18**, paraformaldehyde and the appropriate 4-(2-cyclic-aminoethyl)-piperidine [125]. The incorporation of 1,2,3,4-tetrahydroisoquinoline (**165a**–**c**) was achieved by Chakravorti et al. by adding paraformaldehyde and 1,2,3,4-tetrahydroisoquinoline to the solution of 8HQ (**1**, **18** or **40**) in EtOH and treating the mixture under reflux for 5 h [126].

**Scheme 10:**
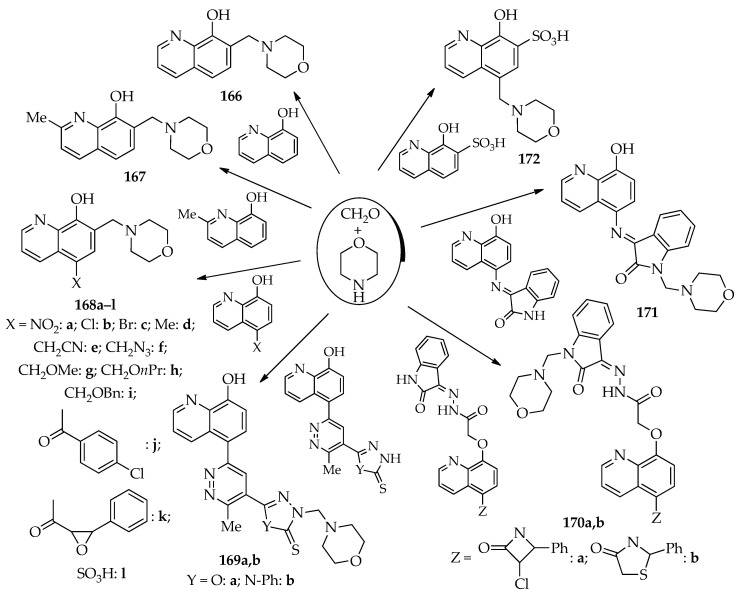
Reaction of 8HQ and substituted 8HQs, CH_2_O, and morpholine.

In addition to piperidine, morpholine, too, was applied rather frequently in the Mannich reaction (Scheme 10). The simplest core in this framework is **166**, which was disclosed by Grzycka and Miłkowska [127]. Similar to **155a**, 8-hydroxyquinaldine **167** was also described by Rose et al. [120]. Petrow and Sturgeon prepared **168a** by treating 5-NO_2_-8HQ in EtOH under reflux with formalin (36%) and morpholine [128]. The synthesis of **168b** in a yield of 78% was reported by Burckhalter et al.; however, its synthesis later appeared in several publications [71]. The 5-bromo analogue **168c** was disclosed by Kim et al. [129]. The preparation of **168d**–**l** and **169**–**172** is an extension of synthetic procedures described previously by Edgerton and Burckhalter (**168d**) [108], Himmi et al. (**168e**,**f**) [109], Venkataramani (**168g**,**h**) [109], Xiao et al. (**168i**) [57], Gopalchari and Dhar (**168j**) [114], Möhrle and Schaltenbrand (**168k**) [115], Sen and Kulkarni (**168l**) [116], Abdelmohsen (**169a**,**b**) [97], Madhu et al. (**170a**,**b**) [123,124], Chhajed and Padwal (**171**) [122] and Yanni and Mohharam (**172**) [96].

**Scheme 11:**
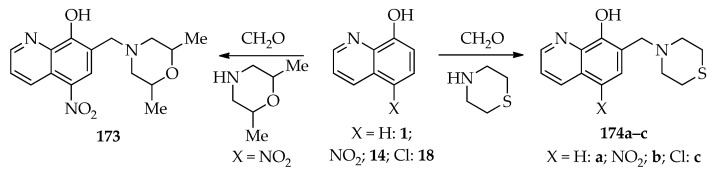
Reaction of 8HQ and 5-substituted 8HQs, CH_2_O and morpholine derivatives.

Morpholine analogues and derivatives, such as thiomorpholine and 2,6-dimethylmorpholine, were also applied in the Mannich reaction. The synthesis of the latter (**173**) was disclosed by Elofsson (Scheme 11) [36]. **174a** was prepared by stirring the components in EtOH (**1**, thiomorpholine, and formalin) for 12 h at r.t. by Zaoui et al. [70]. The synthesis of **174b** and **174c** was carried out by Wangtrakuldee et al. by treating the appropriate 8HQ, CH_2_O and thiomorpholine in dry EtOH at 80 °C for 24 h and isolating the products in yields of 90.6% and 73.7% [33].

In the following, the application of piperazine and *N*-substituted piperazines as secondary amines is demonstrated in the Mannich reaction of 8HQ (**1**) and 5-NO_2_-8HQ (**14**) (Table 7). Another example of the use of MW to carry out aminomethylation of 8HQ is the study by Prati et al. to synthesise **175**, by adding **1** and paraformaldehyde to the dry EtOH solution of piperazine and stirring it at r.t. for 10 min, then treating the mixture at 130 °C for 45 min under MW irradiation [65]. The research group used **175** to synthesise further derivatives, including **178a**, via classical S_N_2 nucleophilic substitution with the appropriate benzyl chloride. Shaw et al., in turn, synthesised **178a** directly from **1**, *N*-benzylpiperazine and paraformaldehyde by stirring the components in dry EtOH at reflux [55]. Shaw et al. used this method successfully for compounds **178b**, **181b**–**o**, **182a**,**b** and **189a**,**b** [55]. Derivatives **176**, **177a**,**c** and **179** were prepared by Faydy et al. by adding the EtOH solution of **1** to the EtOH solution of paraformaldehyde and the amine kept under reflux, then stirring at reflux for 3 h [130,131]. The preparation of **177b** and **185b** was reported by Enquist et al. by reacting CH_2_O and 4-fluorophenylpiperazine under cooling on ice, then adding the resulting precipitation portionwise to **1** or **14** in pyridine at 50 °C [37]. **180** was synthesised by Free et al. [67]. Chen et al. carried out the synthesis of **181a** and **189c** by dissolving the components in dry EtOH and then treating the mixture at reflux for 18–22 h [54]. **183** and **186a**–**c** were obtained by Shterev et al. utilising the same method as applied for **71a** and **163c**–**e** [103]. **184a** and **185a** were prepared by Movrin et al. upon reacting the components in pyridine at 50–60 °C [80]. Yin et al. accomplished the transformation of **14** in pyridine with the appropriate amine at 50 °C for 30–40 min, resulting in **184b**, **185c**–**n** and **187a**–**c** [40]. Sosič et al. also applied pyridine as a solvent to acquire **184c** and **188a**,**b** [60]. Elofsson et al. disclosed the fabrication of **185m** [36].

**Scheme 12:**
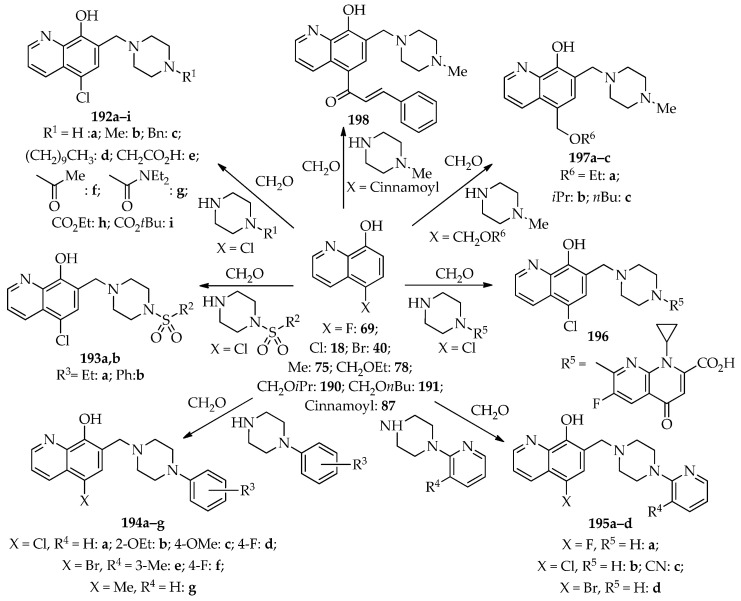
Reactions of 5-Hlg-8HQs, CH_2_O, and piperazine derivatives.

The results of aminoalkylation of 5-halogeno, 5-alkyl and 5-alkoxy 8HQs by piperazine and *N*-substituted piperazines are included in Scheme 12. Prati et al. synthesised **192a** similar to that used for **175** [65]. **192b**–**e**, **192g**,**h** and **193a** were prepared by Burckhalter et al. They also made **192a**, not via Mannich reaction, but by treating **192h** with cc. HCl and then NH_4_OH [71]. Moreover, they prepared **193a** both by the Mannich route and, alternatively, by treating **192a** with ethanesulfonyl chloride. **192f** and **192i** were made by Thinnes et al. by stirring **18**, paraformaldehyde and 1-acyl/Boc-piperazine in the presence of triethylamine in EtOH for 16 h under reflux [132]. Compound **193b** was described by Shaw et al. [55]. The synthesis of **194a**,**b**,**e** was carried out by Enquist et al. using the same method used for **177b** and **185b** [37]. The treatment of **18** in abs. EtOH solution, paraformaldehyde and 1-(4-methoxyphenyl)piperazine in the presence of triethylamine delivered **194c** (Bhat et al.) [45]. **194d**, **194f** and **195b** were disclosed by Elofsson [36]. Among the compounds mentioned previously, **194g** was reported by Edgerton and Burckhalter as well [108]. The syntheses of **195a** and **195d** from **69**/**40**, 1-(pyridin-2-yl)piperazine and formalin (37%) were achieved by Chan et al., stirring the mixture of components at 80 °C for 12 h under a nitrogen atmosphere [68]. The synthesis of compound **195c** was reported by Free et al. [67]. In a rather remarkable study by Fu et al., an 8HQ–ciprofloxacin hybrid was synthesised. The treatment of the dry EtOH solution of **18** with paraformaldehyde and ciprofloxacin as secondary amines for 8 h under reflux gave the desired **196** [133]. Burckhalter and Leib extended their research on **197a**–**c**, whereby the EtOH solution of *N*-methylpiperazine and paraformaldehyde was added to the EtOH solution of 5-alkoxy-8HQ (**75**, **190** or **191**) followed by heating under reflux for 2.5 h [111]. In addition to previous acyl derivatives, 5-cinnamoyl **198** was also described by Schraufstätter and Bock using formalin (30%) as the CH_2_O provider [112].

**Scheme 13:**
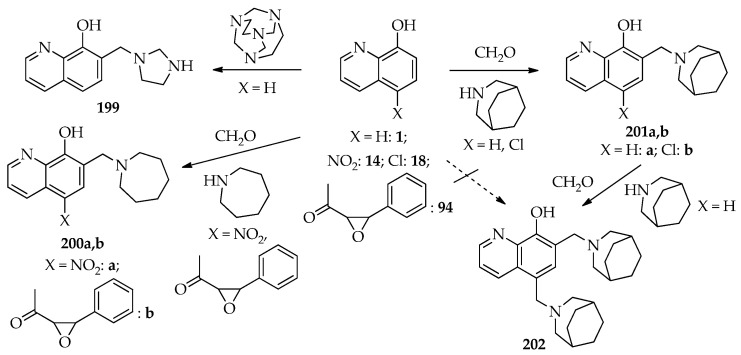
Reaction of 8HQ and 5-substituted 8HQs, CH_2_O and 7-membered cyclic amines.

Scheme 13 depicts the application of seven-membered ring systems. Rivera et al. reported a modification of the Mannich reaction. Instead of using the reactants CH_2_O and an amine, 1,3,6,8-tetraazatricyclo-[4.4.1.1]dodecane (TATD) was added to **1** and isolated from **199**. The process was described as a solvent-free Mannich-type reaction [134]. Shterev et al. extended their investigation to the synthesis of **200a** as well [103]. Möhrle and Schaltenbrand applied azepine similar to Shterev et al., and the synthesis of **200b** was accomplished starting from **94** [115]. Magarian and Nobles used formalin (37%), 3-azabicyclo[3.2.2]nonane and 8HQ (**1** or **14**) in EtOH at reflux to acquire **201a**,**b** [135]. Disubstituted products are not usual in the Mannich reaction, but Magarian and Nobles could achieve the synthesis of **202**; however, it could be synthesised only from **201a** rather than from **1**.

**Scheme 14:**
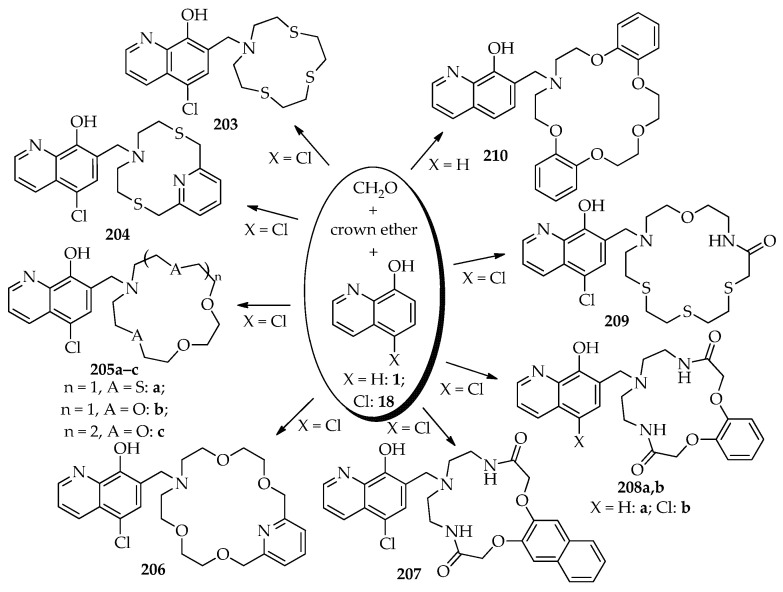
Reaction of 8HQ and 5-Cl-8HQ, CH_2_O, and aza-crown ethers.

An extension of the Mannich reaction is the application of the aza-crown ethers included in Scheme 14. The synthesis of **203**–**205a** was carried out by Aragoni et al., stirring the appropriate aza-crown ether, **18** and paraformaldehyde in benzene under reflux conditions [136,137]. Zhang et al. and Bordunov et al. synthesised **205b**,**c** and **206** via a modified Mannich reaction in two steps. First, the aza-crown ether and paraformaldehyde were stirred in MeOH, followed by evaporation. Subsequently, the formed *N*-methoxymethyl aza-crown ether, as an electrophilic reagent, was stirred with **18** in benzene at reflux temperature, delivering the desired products [138,139,140]. Sharghi and Ebrahimpourmoghaddam treated **18** with paraformaldehyde and macrocyclic ether in the presence of CaCl_2_, and it was found to be efficiently promoting the Mannich reaction. Stirring the mixture without a solvent for 30–60 min at 110 °C, followed by extraction with acetone, gave **207** [141]. In an upcoming study, Sharghi et al. applied an aza-crown ether in the Mannich reaction but perceived a low yield. Therefore, they switched to another, more efficient approach. Specifically, the aza-crown ether was converted to an *N*-methoxymethyl derivative (similar to that by Zhang et al. [138] and Bordunov et al. [139,140]) and then it was stirred with 8HQ (**1** or **18**) and graphite at 100 °C for 10–20 min without solvent, delivering **208a**,**b** [142]. Compound **209** was synthesised by treating azathia-crown ether, **14** and paraformaldehyde in benzene under reflux for 15 h by Song et al. [143]. **210** was furnished from **1**, 4-aza-dibenzo 18-crown-6 ether and paraformaldehyde upon stirring in THF at r.t. for 75 h by Mehta et al. [144].

### 2.2. Syntheses of Mannich Bases Furnishing 2:1:1 Moiety Ratio in the Product (CH_2_O:Amine:8HQ)

#### 2.2.1. Syntheses by Using Primary Amines Delivering Dihidro-1,3-Oxazinoquinolines

Table 8 depicts the reactions of 1 molar equivalent 8HQ derivatives with 1 equivalent alkyl amines and 2 equivalents of formaldehyde. The reasons to acquire an oxazine scaffold are the following: The aim of the synthesis was the benzoxazine core itself, but sometimes it was considered an intermediary product or a side-product—during the synthesis of furnishing open-chain Mannich derivatives (see Table 2, **30a**–**c**). The formation of **212a** was achieved from **1**, aniline and paraformaldehyde by Ma et al. [145]. In order to prepare **212b** and **214a**–**f**, March et al. stirred the appropriate 8HQ (**1** or 2-trifluoromethyl-8HQ—**210**), paraformaldehyde and several amines in 50% benzene–EtOH at reflux for 2 h [146]. Page et al. synthesised **212b** as well as **212c**,**d** as starting compounds for their further transformation via reductive cleavage to obtain asymmetric acyclic aminomethyled 8HQs. Compounds 7-ethylmethyl and 7-benzylmethyl aminomethylquinolin-8-ols (similar derivatives are listed in Table 4) were efficiently prepared from **212b** and **212c** [147]. In addition to **39e** with the 7-aminomethyl side chain, **213a**,**c**,**e**,**g**–**k** and **215a**,**b** were also synthesised by Gianni et al. They, however, applied different conditions by treating the corresponding amine and paraformaldehyde under reflux in EtOH for 6 h, followed by cooling to r.t., adding **1** or **211** and stirring at r.t. for 12 h [89]. Manetti et al. used identical conditions as used by Gianni et al., delivering **213b**,**d**,**f** [73]. Si et al. treated paraformaldehyde and the appropriate amine in 1,4-dioxane at 75 °C for 30 min, and then **58** was added and stirred at 75 °C overnight, producing **216a**–**g** [148].

**Scheme 15:**
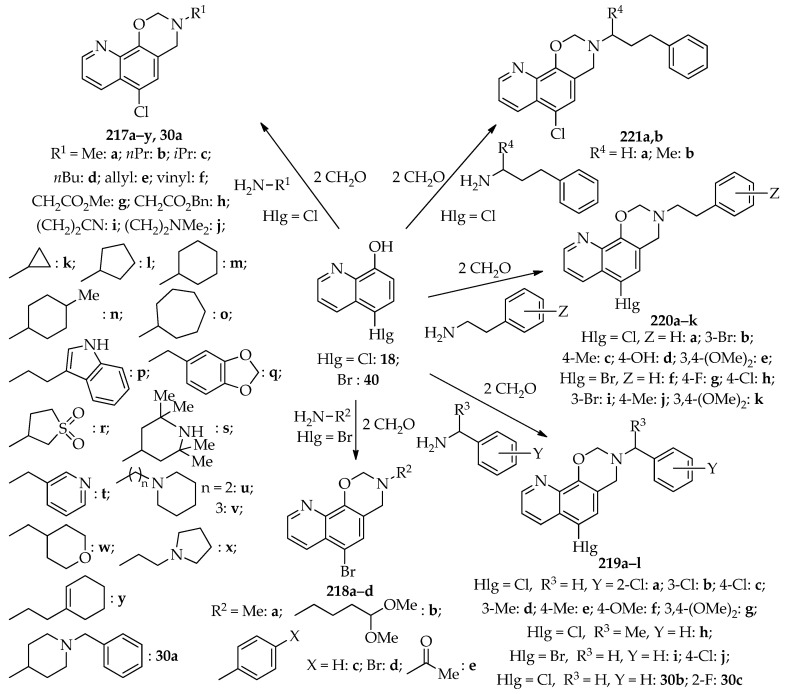
Reaction of 5-Hlg-8HQs, CH_2_O, and primary amines.

Many attempts were made to transform 5-Cl- and 5-Br-8HQ into oxazine derivates (Scheme 15). Fu et al. efficiently applied the same method and conditions used in the synthesis of the 8HQ–ciprofloxacin hybrid (**196**) and carried out the synthesis of **217a**–**c**, **217e**–**k** and **217v**–**x** [133]. Other *N*-alkyl derivatives were described by Kim et al. (**217d**) [129], Fiorentino et al. (**217l**, **219h**) [149], Olaleye et al. (**217m**) [150], Gianni et al. (**217n**–**p**) [89], March et al. (**217q**) [146] and Ratan et al. (**217r**–**u**) [151]. Bowlin and co-workers carried out the synthesis of **217y** in a solvent mixture of benzene and EtOH [152]. **30a** was synthesised by Ahn et al. applying **18**, 4-amino-1-benzylpiperidine and 2 molar equivalents of paraformaldehyde. Note that the formation of **33a** (Table 2) was also observed [63]. **218a**, **218c**–**e** and **219i** were studied by Mordarski and Chylińska [153]. **218b**, **219j**, **220b**–**k** and **221a**,**b** were synthesised via the method used for **216a**–**g** by Si et al. [148]. March et al. carried out the synthesis of **30b** and **219b**–**e** in 50% benzene–EtOH, similarly to how **212b** and **214a**–**f** were prepared (Table 8) [146]. Identical and resembling derivatives (**30b**, and **219a**,**f**,**g**) were furnished by treating the components (**14**, appropriate benzylamine and paraformaldehyde in a molar ratio of 1:1:2) in EtOH in the presence of KOH at reflux for 1 h by Combes and Mesnier [88]. **30c** was described by Szakács et al., and its open-chain product **39b** was transformed by means of HCl/EtOH (22%) (Table 2) [48]. **220a** was included in the study by De La Fuente et al. [154].

#### 2.2.2. Syntheses by Using Primary Amines Furnishing Azabicyclo Derivatives

**Scheme 16:**
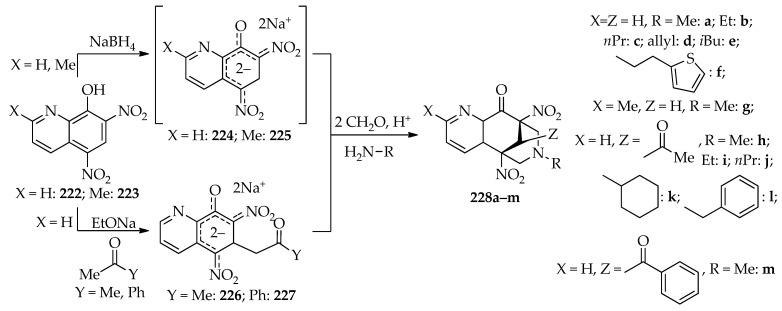
Reaction of 5,7-dinitro-8HQs, CH_2_O and primary amines.

Scheme 16 depicts an intriguing extension of the Mannich reaction, whereas the incorporated CH_2_O and the amine form a bridge between positions 5 and 7. The transformation of 5,7-dinitro-8HQ (**222**) and its 2-Me derivative (**223**) was carried out by Yakunina et al. and Medvedeva et al., resulting in several structural analogues of cytisine [155,156,157]. **228a**–**g** were obtained by treating **222** or **223** with NaBH_4_ in a DMF–EtOH mixture for 10 min under cooling, resulting in the intermediary hydride σ^H^-adducts (**224** and **225**). Subsequently, formalin (32%) and the appropriate amine in water were added to the reaction mixture and then it was acidified with 20% phosphoric acid. From the reaction of **222** with acetone and sodium ethoxide, the Janovszky σ-adduct **226** was isolated, which was transformed to **228h**–**l** with formalin (32%) and the appropriate amine. **228m** was synthesised in a similar manner; the only modification was that acetophenone was applied instead of acetone, and the intermediary σ-adduct was compound **227**.

### 2.3. Syntheses of Mannich Bases Furnishing 2:1:2 Moiety Ratio in the Product (CH_2_O:Amine:8HQ)

#### 2.3.1. Syntheses by Using Primary and Cyclic Secondary Amines

**Scheme 17:**
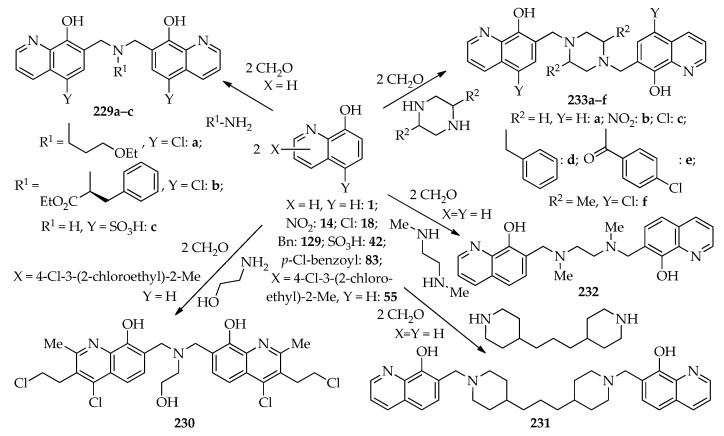
Reaction of 8HQs, CH_2_O and primary amines.

Some unusual Mannich bases are included in Scheme 17. Fu et al. applied the method described previously (Scheme 11) to form **229a**,**b** as well [133]. **229c** was furnished by treating **42** with formalin (37%) in 7% aqueous ammonia at 90 °C for 40 min by Matsumura et al. [158]. When the synthesis of **62b** from **55** was performed by Ozawa and Shibuya, the formation of side-product **230** was observed. By reducing the molar equivalent of ethanolamine, **230** became the main isolable product [94]. Shebab et al. applied 1,3-di(piperidine-4-yl)propane and formalin (37%) to treat **1** in EtOH at r.t., which led to the formation of **231** [39]. **232** was obtained from the reaction of **1**, *N*,*N*’-dimethylethylenediamine and formalin (37%) in EtOH at 50 °C by Zaoui et al. [70]. **233a** was furnished via stirring **1**, paraformaldehyde and piperazine in EtOH at reflux in the presence of HCl by Raj et al. [159] The preparation of **233b** was performed in a mixture of pyridine and DMF by Movrin et al. [80]. **233c** and **233f** were synthesised via stirring the starting components (**14**, paraformaldehyde and piperazine or *trans*-2,5-dimethylpiperazine) in EtOH at reflux by Burckhalter et al. [71]. **233d** and **233e** were obtained by Gopalchari and Dhar by applying paraformaldehyde as the CH_2_O source and treating the reaction in EtOH at reflux for 3–4 h [114].

#### 2.3.2. Syntheses by Using Diaza-Crown Ethers

**Scheme 18:**
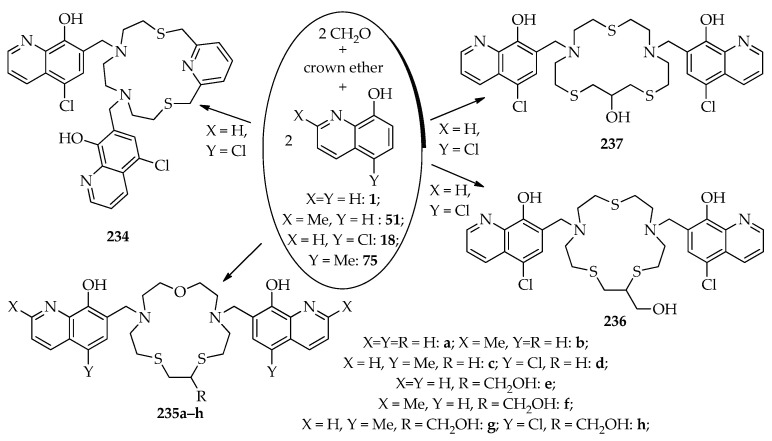
Reaction of 8HQ and substituted 8HQs, CH_2_O and diaza-crown ethers.

When two secondary aliphatic amines are included in a crown ether (for instance, diazadithia-15-crown-5, diazatrithia-15-crown-5, etc.), their application leads to compounds depicted on Scheme 18. Shamsipur et al. stirred **18**, paraformaldehyde and a macrocyclic amine in benzene at reflux, resulting in **234** [160]. Identical conditions were applied to synthesise **235a**–**d** (Bradshaw et al. [161]) and **235e**–**h** (Song et al. [162]). Bronson et al. prepared **236** and **237** by stirring diaza-crown ethers and paraformaldehyde in MeOH overnight, followed by the removal of MeOH. Subsequently, benzene and **18** were added, and the mixture was treated at reflux for 24 h [163].

**Scheme 19:**
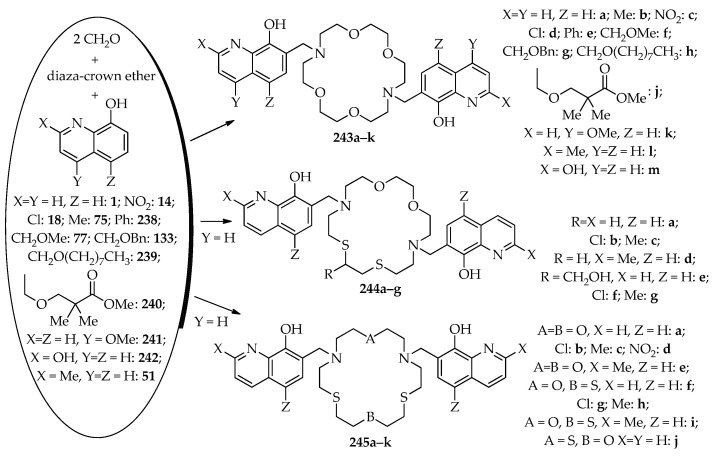
Reaction of 8HQ and substituted 8HQs, CH_2_O and diaza-crown ethers.

Diaza-18-crown-6, diazadithia-18-crown-6 and other S-containing diaza-crown ethers were also used in Mannich reactions (Scheme 19). Su et al. prepared **243a**–**c** and **243m** by means of the reaction of the 8HQ derivative, paraformaldehyde and an appropriate crown ether in anhydrous toluene under reflux conditions [164]. **243d** was synthesised by Bordunov et al. under the same conditions as **205c** (Scheme 14) [139]. Farruggia et al. used microwave irradiation (600 W for 2–4 h) to optimise the preparation of **243e**–**l** and efficiently carried out the syntheses in both toluene and 1,4-dioxane [165]. In addition to compounds described previously, **244a**–**d**, **245a**–**c**, and **245e**–**i** were furnished by Bradshaw et al. [161], **244e**–**g** and **245d** by Song et al. [143,162] and **245j** by Bronson et al. [163].

**Scheme 20:**
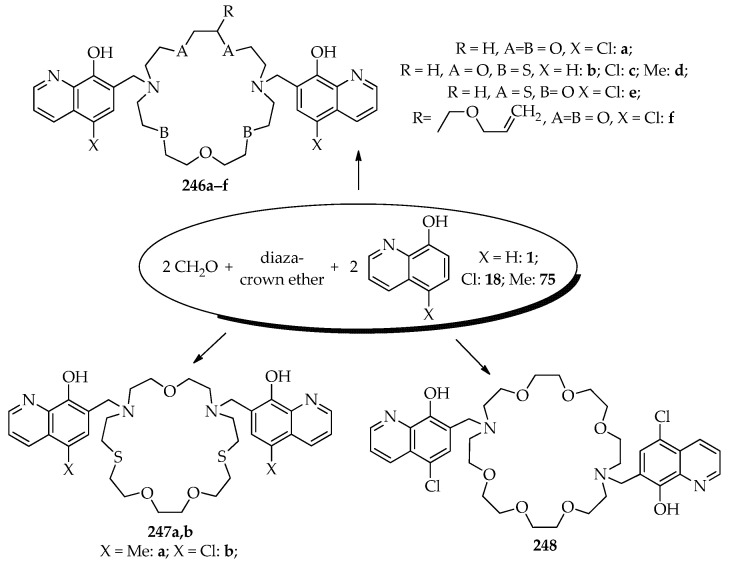
Reaction of 5-substituted 8HQs, CH_2_O and diaza-crown ethers.

Further extensions to diaza-21-crown-7, its sulphur analogues and diaza-24-crown-8 ether were performed by Song et al. and Bordunov et al. as depicted in Scheme 20, including **246a** (Song et al.) [166], **246b**–**d** (Song et al.) [143], **246e**, **247a**,**b** (Song et al.) [162] as well as **246f** and **248** (Bordunov et al.) [139].

### 2.4. Syntheses of Mannich Bases Furnishing Products in Miscellaneous Ratios

#### Syntheses by Furnishing (Methylene)bisproducts with 2:2:1 Ratio in the Product (CH_2_O:Amine:8HQ)

**Scheme 21:**
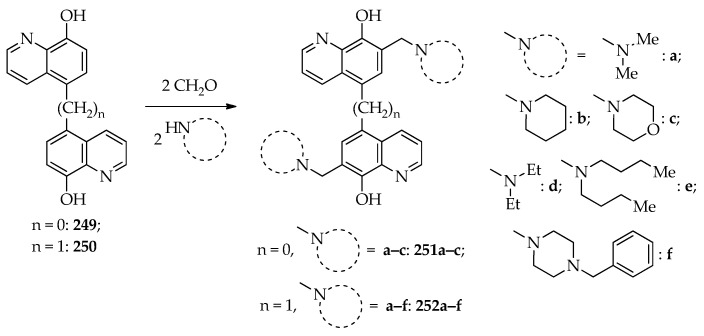
Reaction of bis and methylenebis 8HQs, CH_2_O and secondary amines.

In general, diaminomethylation in the Mannich reaction is not a common occurrence, although compound **202** is a noteworthy exception (Scheme 13). On the other hand, it is relatively simple to obtain disubstitution when the starting compound is a bis or methylenebis compound (Scheme 21). Gopalchari and Dhar first synthesised **249** and **250**, and from these derivatives, **251a**–**c** and **252a**–**d** were prepared by means of different secondary amines and paraformaldehyde [167]. **252e** was furnished by Xie et al. by treating **250** with formalin (37%) and dibutylamine in DMF under reflux for 4 h [168]. Abdelhameed et al. reacted **250** with formalin (37%) and *N*-benzylpiperazine in MeOH at r.t. for 24 h, resulting in the formation of **252f** [169].

**Scheme 22:**
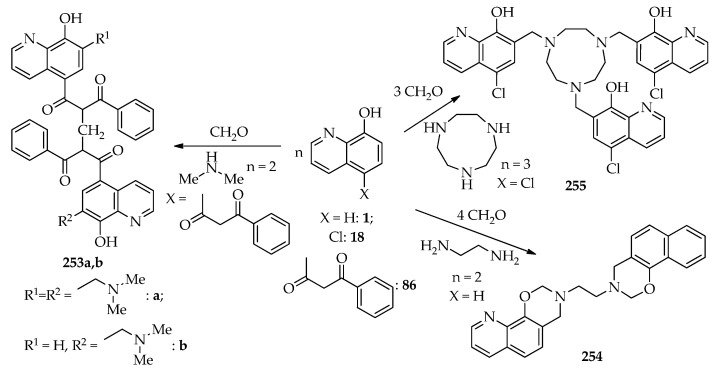
Reaction of 8HQ and 5-Cl-8HQ, CH_2_O, and different amines.

Scheme 22 depicts bis- and trisproducts starting from “mono” compounds. **253a**,**b** were obtained under conventional Mannich conditions by Möhrle and Schaltenbrand. The interesting fact is that the side chains were involved in the reaction; therefore, methylenebis compounds were isolated rather than the 7-aminomethylated product, in contrast to compound **104** (Table 5) [170]. In the reaction of **254**, formalin (37%), ethylenediamine and **1**, with ratios of 4:1:2, were dissolved in 1,4-dioxane under stirring at r.t. for 4 h by Rivera et al. [134]. Bencini et al. carried out the transformation of **255** with 1,4,7-triazacyclononane and paraformaldehyde in toluene at 90 °C with a yield of 62% [171].

## 3. Conclusions

The past milestones and current trends in the Mannich reaction of 8HQ have been reviewed. Transformations were performed utilising various amines and formalin or paraformaldehyde as the CH_2_O source. In some cases, the Mannich reaction was carried out without a solvent; however, it is a general trend to perform these reactions in a solvent or a mixture of solvents, primarily in EtOH. Pyridine, benzene (mainly in the case of aza-crown ethers), MeOH, DMF, toluene, 1,4-dioxane, and their mixtures were also utilised. Both the order of the applied amine and the molar equivalents of the reactants were crucial. The reactions took place at position 7 of the 8HQ core, except when the 7-position was substituted and when the structure had a more reactive position (1,3-dicarbonyl side chain or (thio)amide-containing ring).

The aminomethylated 8HQ derivatives possess several biological properties and can affect pharmacological targets, including pathogenic microorganisms, cancer cells and central nervous system targets.

Research on aminomethylation via the Mannich reaction over more than a century has generated a series of versatile compounds with broad structural diversity. The ongoing advancement of the area undoubtedly draws the attention of researchers and demonstrates that there is much more to discover in the Mannich chemistry of 8HQs.

## Figures and Tables

**Table 1:** Reaction of 8HQ, CH_2_O, and primary amines.

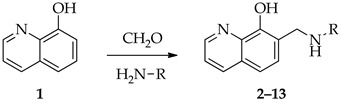			
Compound	–R	Conditions	Refs.
**2**	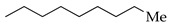	EtOH, reflux (78 °C), 1 h; Yield: 98%	[70]
**3a**,**b**	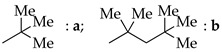	**a**: Benzene, reflux, 30 min; Yield: n.d. **b**: Neat, 104 °C, 1 h; Yield: n.d.	[71]
**4**	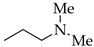	EtOH, reflux, 90 min; Yield: 90%	[72]
**5a**,**b**	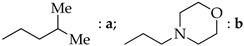	EtOH, reflux, 4 h; Yield: n.d.	[46]
**6a**–**c**	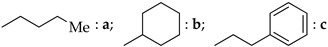	MeOH, reflux, 12 h; Yield: **a**: 13%; **b**: 3%; **c**: 31%	[73]
**7**	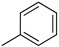	EtOH, r.t. 5 min → 120 °C, 12 h; Yield: 52%	[47]
**8**	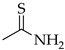	DMF, 60 °C, 6 h; Yield: 92.75%	[74]
**9**	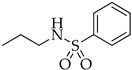	EtOH, r.t.→reflux, 18–22 h; Yield: n.d.	[55]
**10a**–**c**	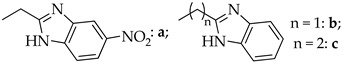	EtOH, r.t. → 0 °C; Yield: n.d.	[75]
**11a**–**d**	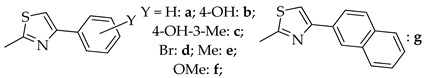	EtOH, reflux, HCl; Yield: **a**: 65%; **b**: 65%; **c**: 68%; **d**: 46%; **e**: 52%; **f**: 54%; **g**: 70%	[76,77,78]
**12a**–**m**	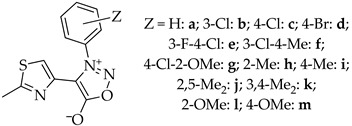	EtOH, reflux, 8 h, HCl; Yield: **a**: 85%; **b**: 85%; **c**: 80%; **d**: 85%; **e**: 75%; **f**: 75%; **g**: 65%; **h**: 75%; **i**: 82%; **j**: 65%; **k**: 65%; **l**: 70%; **m**: 70%	[79]
**13a**–**g**	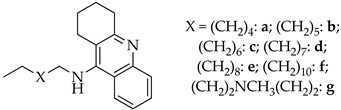	EtOH, 90 °C, 6 h → r.t. 18 h; Yield: **a**: 45%; **b**: 38%; **c**: 23%; **d**: 38%; **e**: 20%; **f**: 22%; **g**: 25%	[64]

**Table 2:** Reaction of 5-Cl-8HQ, CH_2_O and primary amines.

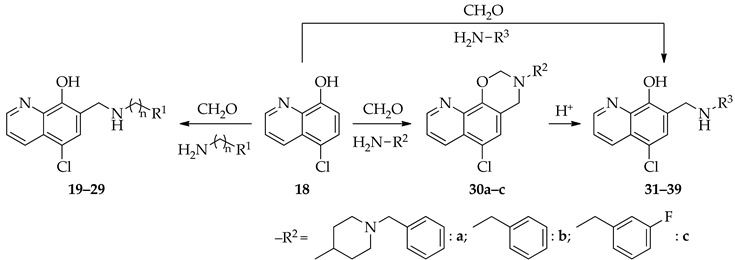						
Compound	n	–R^1^	Refs.	Compound	–R^2^	Refs.
**19a**,**b**	2: **a**, 3: **b**	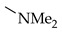	[71,82]	**31**	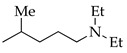	[82]
**20a**–**e**	2: **a**, 3: **b**; 4: **c**; 5: **d**; 6: **e**	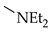	[71,82,85]			
**21**	3	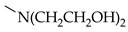	[82]	**32**	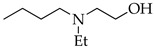	[82]
**22**	3	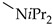	[82]	**33a**,**b**	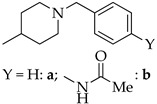	[63]
**23a**–**c**	3: **a**; 4: **b**; 5: **c**	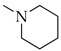	[82,85]			
**24a**,**b**	3: **a**; 5: **b**	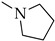	[82]			
**25**	3	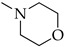	[82]	**34**	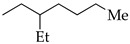	[85]
**26**	3	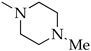	[85]	**35a**,**b**	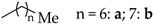	[85]
				**36**	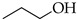	[85]
**27a**,**b**	3	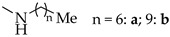	[82]	**37**	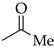	[86]
**28**	3	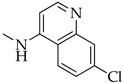	[42]	**38**	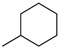	[48]
**29a**–**e**	1	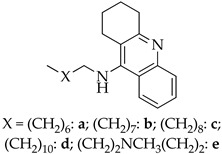	[64]	**39a**–**e**	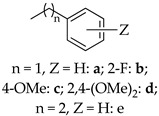	[48,87,89]

**Table 3:** Reaction of 8HQ derivatives, CH_2_O and primary amines.

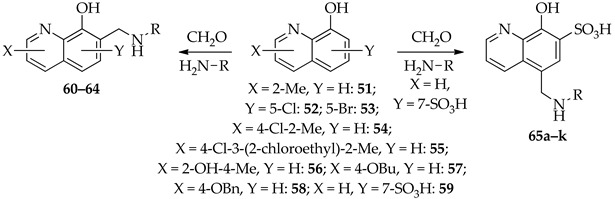				
Compound	X and Y	–R	Conditions	Refs.
**60a**–**e**	2-Me	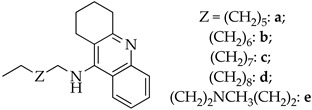	EtOH, 90 °C, 6 h → r.t. 18 h; Yield: **a**: 33%; **b**: 35%; **c**: 35%; **d**: 35%; **e**: 27%	[64]
**61a**–**c**	2-Me: **a**; 5-Cl-2-Me: **b**; 5-Br-2-Me: **c**	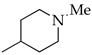	EtOH, reflux; Yield: n.d.	[93]
**62a**,**b**	4-Cl-2-Me: **a**; 4-Cl-3-(2-chloroethyl)-2-Me: **b**	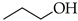	EtOH, reflux; Yield: **a**: 87%; **b**: 40%	[94]
**63a**,**b**	4-OBu: **a**; 4-OBn: **b**	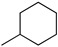	EtOH, reflux, 12 h; Yield: **a**: 8%; **b**: 53%	[73]
**64a**,**b**	2-OH-4-Me	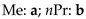	EtOH, reflux, 9 h; Yield: **a**: 58%; **b**: 50%	[95]
**65a**–**k**	7-SO_3_H	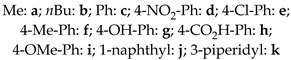	EtOH, reflux, 30–50 h; Yield: 60–70%	[96]

**Table 4:** Reaction of 8HQ and 5-substituted 8HQs, CH_2_O and secondary acyclic amines.

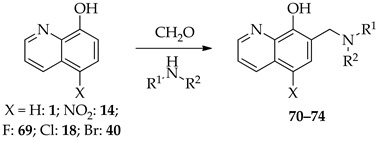				
Compound	X	–R^1^, –R^2^	Conditions	Refs.
**70a**–**f**	H	R^1^ = R^2^ = Me: **a**; Et: **b**; CH_2_CO_2_H: **c**; *n*Pr: **d**; *n*Bu: **e**; *n*-hexyl: **f**; cyclohexyl: **g**; 2-ethylhexyl: **h**; *n*-octyl: **i**; *n*-dodecyl: **j**; *n*-octadecyl: **k**; *iso*-dodecenyl: **l**; Ph: **m**; octylphenyl: **n**; Bn: **o**; 3-(octyloxy)propoxy: **p**; R^1^ = *n*-octyl, R^2^ = Ph: **q**; 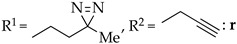 : **r**	**a**: EtOH, reflux, 2 h; Yield: 74% **b**: EtOH, r.t., 1 h → reflux, 5 h; Yield: 55% **c**: DMF, reflux, 24 h; Yield: 27% **d**: EtOH, r.t., 1 h → reflux, 3 h; Yield: 65% **e**–**q**: MeOH, 20 °C, 1 h→reflux, 3 h; Yield: n.d. **r**: EtOH, reflux 60 °C, 48 h; Yield: 51%	[48,98,99,100,101,102]
**71a**–**i**	NO_2_	R^1^ = R^2^ = Me: **a**; Et: **b**; *n*Pr: **c**; *i*Pr: **d**; (CH_2_)_2_OH: **e**; cyclohexyl: **f**; R^1^ = Me, R^2^ = cyclohexyl: **g**; R^1^ = Me, R^2^ = CH_2_CN: **h**; R^1^ = Bn, R^2^ = CH_2_CN: **i**	**a**: pyridine:EtOH 1:1, reflux; Yield: n.d. **b**: EtOH, reflux, 90 min; Yield: 83% **c**: EtOH, reflux 80 °C, 24 h; Yield: 14% **d**, **e**: pyridine, 50–60 °C; Yield: 74–84% **f**: EtOH, reflux 80 °C, 24 h; Yield: 19% **g**: n.d. **h**, **i**: pyridine, 60 °C; Yield: 70%/74%	[36,40,41,60,80,103,104]
**72**	F	R^1^ = R^2^ = Et	EtOH, r.t., 30 min; Yield: 42%	[105]
**73a**–**l**	Cl	R^1^ = R^2^ = Me: **a**; Et: **b**; *n*Pr: **c**; *n*Bu: **d**; (CH_2_)_2_OH: **e**; CH_2_CO_2_Et: **f**; CH_2_CN: **g**; R^1^ = Me, R^2^ = (CH_2_)_2_NEt_2_: **h**; R^1^ = Me, R^2^ = (CH_2_)_3_NEt_2_: **i**; R^1^ = Et, R^2^ = (CH_2_)_2_NEt_2_: **j**; R^1^ = Et, R^2^ = (CH_2_)_3_NEt_2_: **k**; R^1^ = Et, R^2^ = (CH_2_)_2_OH: **l**;	**a**: EtOH, reflux, 90 min; Yield: 75% **b**: EtOH, reflux, 90 min; Yield: 74% **c**, **d**: EtOH, reflux, 2 h/30 min; Yield: n.d. **e**: EtOH, reflux, 90 min; Yield: 70% **f**: EtOH, reflux, 2 h; Yield: 79% **g**: EtOH, reflux, 3 h; Yield: 42% **h**–**k**: EtOH, reflux, 90 min; Yield: n.d. **l**: EtOH, reflux, 90 min; Yield: 80%	[71,82,85,106,107]
**74a**,**b**	Br	R^1^ = R^2^ = Et: **a**; R^1^ = Me, R^2^ = Et: **b**	**a**: EtOH, reflux, 90 min; Yield: 51% **b**: EtOH, reflux, 1 h; Yield: n.d.	[106,107]

**Table 5:** Reaction of 5-substituted 8HQs, CH_2_O and secondary acyclic amines.

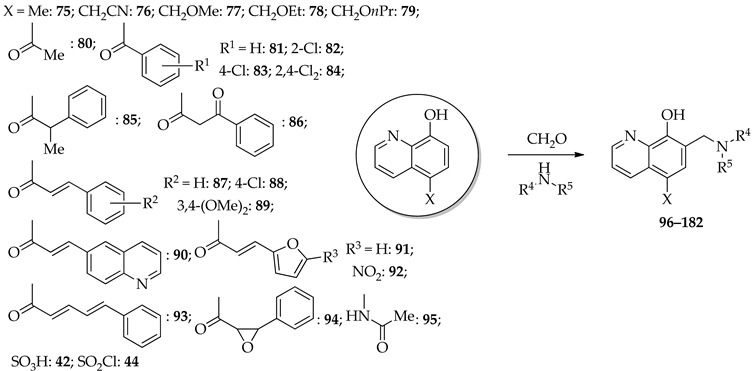							
Compound	X	–R^4^, –R^5^	Refs.	Compound	X	–R^4^, –R^5^	Refs.
**96a**,**b**	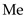	R^4^ = R^5^ = Et: **a**; R^4^ = Et, R^5^ = (CH_2_)_2_OH: **b**	[108]	**106a**,**b**	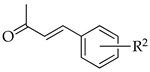	R^2^ = 4-Cl, R^4^ = R^5^ = Me: **a**; R^2^ = 3,4-(OMe)_2_, R^4^ = R^5^ = Me: **b**	[112]
**97**	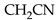	R^4^ = R^5^ = Bn	[109]				
**98**	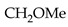	R^4^ = R^5^ = (CH_2_)_2_OH	[109]				
**99**	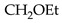	R^4^ = R^5^= Et	[111]	**107**	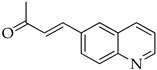	R^4^ = R^5^ = Me	[112]
**100**	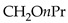	R^4^ = R^5^ = (CH_2_)_2_OH	[109]	**108a**,**b**	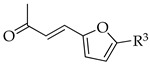	R^3^ = H, R^4^ = R^5^ = Me: **a**; R^3^ = NO_2_, R^4^ = R^5^ = Me: **b**	[112]
**101a**,**b**	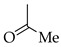	R^4^ = R^5^ = Me: **a**; R^4^ = (CH_2_)_2_CN, R^5^ = C_12_H_25_: **b**	[112,113]	**109**	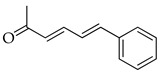	R^4^ = R^5^ = Me	[112]
**102a**–**f**	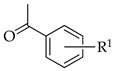	R^1^ = H, R^4^ = R^5^ = Me: **a**; R^1^ = H, R^4^ = Et, R^5^ = (CH_2_)_2_NEt_2_: **b**; R^1^ = 2-Cl, R^4^ = R^5^ = Me: **c**; R^1^ = 4-Cl, R^4^ = R^5^ = Me: **d**; R^1^ = 2,4-Cl_2_, R^4^ = R^5^ = Me: **e**; R^1^ = 4-Cl, R^4^ = R^5^ = Et: **f**	[112,114]				
**103**	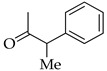	R^1^ = R^2^ = Me	[112]	**110a**,**b**	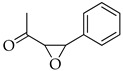	R^4^ = R^5^ = Me: **a**; Et: **b**	[115]
**104**	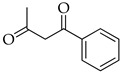	R^1^ = R^2^ = Me	[112]	**111**	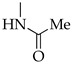	R^4^ = R^5^ = Et	[104]
**105a**–**h**	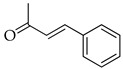	R^4^ = R^5^ = Me: **a**; Et: **b**; *n*Bu: **c**; (CH_2_)_2_OH: **d**; (CH_2_)_2_NEt_2_: **e**; R^4^ = Me, R^5^ = Bn: **f**; R^4^ = Me, R^5^ = C_12_H_25_: **g**; R^4^ = Et, R^5^ = (CH_2_)_2_NEt_2_: **h**	[112]	**112a**–**f**	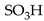	R^4^ = R^5^ = Me: **a**; Et: **b**; *n*Pr: **c**; *n*Bu: **d**; *i*Bu: **e**; (CH_2_)_2_OH: **f**	[116]
				**113**	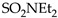	R^4^ = R^5^ = Et	[92]

**Table 6:** Reaction of 8HQ and 5-substituted 8HQs, CH_2_O and piperidine.

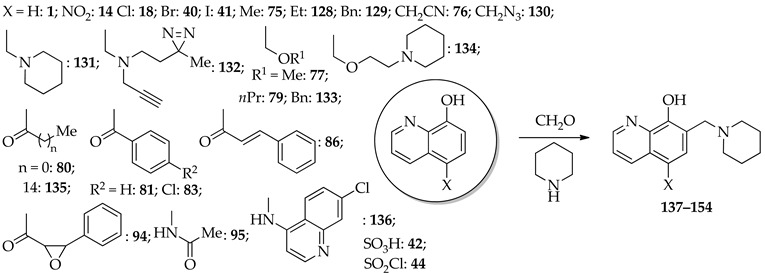							
Compound	X	Conditions	Refs.	Compound	X	Conditions	Refs.
**137**	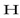	EtOH, reflux, 6 h; Yield: n. d.	[5]	**148a**–**d**	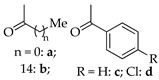	EtOH, reflux, 1–4 h; Yield: **a**: 67%; **b**: 81%; **c**: 54% **d**: n.d.	[108,112]
**138**	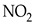	EtOH, heat; Yield: 83%	[104]				
**139**	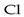	EtOH, reflux, 1.5 h; Yield: 80%	[71]				
**140**	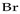	EtOH, reflux, 1 h; Yield: 65%	[111]	**149**	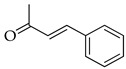	EtOH, reflux, 8 h; Yield: n. d.	[112]
**141**	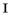	EtOH, reflux; Yield: 60%	[90]				
**142a**–**c**	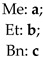	EtOH, reflux, 1–2 h; Yield: **a**: 97%; **b**: 95%; **c**: 56%	[108]	**150**	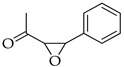	n. d.; Yield: 67–90%	[115]
**143a**,**b**	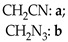	EtOH, reflux, 4 h; Yield: **a**: 78%; **b**: 81%	[109]				
**144**	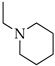	Neat conditions, heat, 3 h; Yield: 90%	[111]	**151**	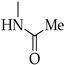	EtOH, reflux, 1 h; Yield: 92%	[104]
**145**	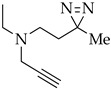	EtOH, r. t., 3 days; Yield: 68%	[48]	**152**	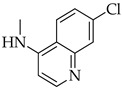	EtOH, reflux, 2 h; Yield: 93%	[104]
**146a**–**c**	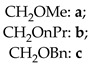	**a**, **b**: n. d. **c**: EtOH, reflux, 4 h; Yield: n. d.	[57,109]	**153**	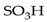	EtOH, reflux, 6 h; Yield: 80%	[116]
**147**	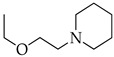	EtOH, reflux, 3 h; Yield: 88%	[111]	**154**	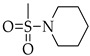	EtOH, reflux, 30–40 h; Yield: 70%	[92]

**Table 7:** Reaction of 8HQ and 5-NO_2_-8HQ, CH_2_O and piperazine derivatives.

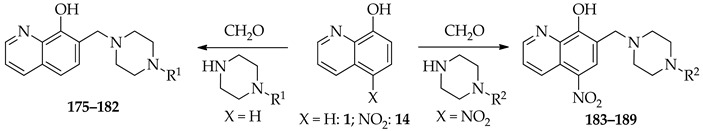					
Compound	–R^1^	Refs.	Compound	–R^2^	Refs.
**175**	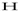	[65]	**183**	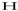	[103]
**176**	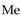	[130]	**184a**–**c**	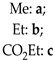	[40,60,80]
**177a**–**c**	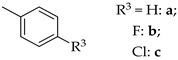	[37,130,131]	**185a**–**m**	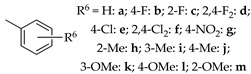	[36,37,40,80]
**178a**,**b**	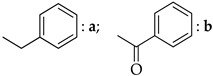	[55]			
**179**	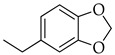	[131]	**186a**–**c**	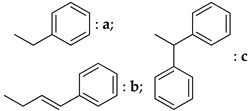	[103]
**180**	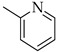	[67]			
**181a**–**o**	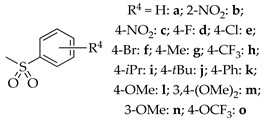	[54,55]	**187a**–**c**	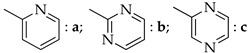	[40]
			**188a**,**b**	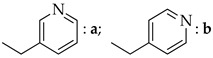	[60]
**182a**,**b**	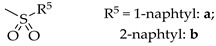	[55]	**189a**–**c**	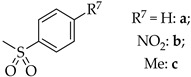	[54,55]

**Table 8:** Reaction of 8HQ and substituted 8HQs, CH_2_O and primary amines.

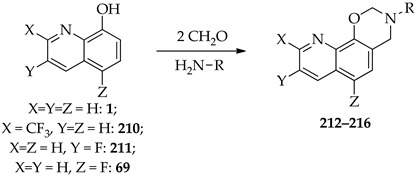				
Compound	X/Y/Z	–R	Conditions	Refs.
**212a**–**d**	X = H Y = H Z = H	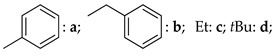	**a**: n.d. **b**: Benzene:EtOH 1:1, reflux 2 h; Yield: 18% **c**, **d**: n.d.	[145,146,147]
**213a**–**h**	X = H Y = H Z = H	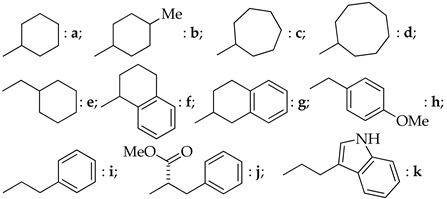	EtOH, reflux 6 h → r.t. 12 h; Yield: **a**: 35%; **b**: 18%; **c**: 51%; **d**: 15%; **e**: 48%; **f**: 51%; **g**: 87%; **h**: 48%; **i**: 30%; **j**: 21%; **k**: 10%	[73,89]
**214a**–**f**	X = CF_3_ Y = H Z = H	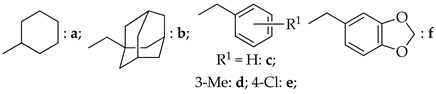	Benzene:EtOH 1:1, reflux 2 h; Yield: **a**: 44%; **b**: 47%; **c**: 50%; **d**: 22%; **e**: 24%; **f**: 15%	[146]
**215a**,**b**	X = H Y = F Z = H	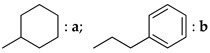	EtOH, reflux 6 h → r.t. 12 h; Yield: **a**: 50%; **b**: 55%	[89]
**216a**–**g**	X = H Y = H Z = F	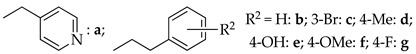	1,4-dioxane, 75 °C; Yield: **a**: 35.0%; **b**: 52.6%; **c**: 68.7%; **d**: 62.9%; **e**: 34.3%; **f**: 67.6%; **g**: 81.6%	[148]

## Data Availability

Not applicable.
